# Bayesian spatial modeling of childhood overweight and obesity prevalence in Costa Rica

**DOI:** 10.1186/s12889-023-15486-1

**Published:** 2023-04-05

**Authors:** Mario J. Gómez, Luis A. Barboza, Paola Vásquez, Paula Moraga

**Affiliations:** 1 Computer, Electrical and Mathematical Sciences and Engineering Division, King Abdullah University of Science and Technology (KAUST), Thuwal, Saudi Arabia; 2 Centro de Investigación en Matemática Pura y Aplicada-Escuela de Matemática, Universidad de Costa Rica, San José, Costa Rica; 3 Centro de Investigación en Matemática Pura y Aplicada, Universidad de Costa Rica, San José, Costa Rica

**Keywords:** Childhood, Overweight, Obesity, Bayesian, Spatial, Multinomial, Costa Rica

## Abstract

**Background:**

Childhood overweight and obesity levels are rising and becoming a concern globally. In Costa Rica, the prevalence of these conditions has reached alarming values. Spatial analyses can identify risk factors and geographical patterns to develop tailored and effective public health actions in this context.

**Methods:**

A Bayesian spatial mixed model was built to understand the geographic patterns of childhood overweight and obesity prevalence in Costa Rica and their association with some socioeconomic factors. Data was obtained from the 2016 Weight and Size Census (6 - 12 years old children) and 2011 National Census.

**Results:**

Average years of schooling increase the levels of overweight and obesity until reaching an approximate value of 8 years, then they start to decrease. Moreover, for every 10-point increment in the percentage of homes with difficulties to cover their basic needs and in the percentage of population under 14 years old, there is a decrease of 7.7 and 14.0 points, respectively, in the odds of obesity. Spatial patterns show higher values of prevalence in the center area of the country, touristic destinations, head of province districts and in the borders with Panama.

**Conclusions:**

Especially for childhood obesity, the average years of schooling is a non-linear factor, describing a U-inverted curve. Lower percentages of households in poverty and population under 14 years old are slightly associated with higher levels of obesity. Districts with high commercial and touristic activity present higher prevalence risk.

## Background

According to the World Health Organization (WHO) [1], the combined prevalence of overweight and obesity among children and adolescents aged 5-19 worldwide, has risen from 4% in 1975 to 18% in 2016, becoming one of the most significant global public health challenges of the 21st century [2]. Besides the immediate health effects like breathing difficulties, increased risk of fractures, hypertension, early markers of cardiovascular disease, and insulin resistance, this condition tends to persist into adulthood, deriving in a higher chance of premature death and disability [1]. In Costa Rica, the latest Weight and Size Census carried out in 2016 showed that overweight and obesity combined prevalence has reached a concerning 34.5% among children in school age (6-12) [3] for which the need to give an effective and timely response has been recognized [4].

The key determinant of weight gain in children is simply the surpassing of caloric consumption over expenditure, however, family and community related factors affect individual habits and behaviors that leads to this imbalance [5, 6]. Recent research has focused on the study of these kind of drivers in a broader ecological context [7, 8] in which population health and environment are interdependent in both micro (e.g., homes, schools) and macro (e.g., food distribution, urban/rural development) levels [9]. The intrinsic geographic nature of this approach and the resulting benefits of knowing where interventions are needed and if they were successful (critical in developing countries where resources are scarce) have encouraged the use of spatial statistics in obesity research and public health policy implementation [10].

In spatial statistical modeling, characterizing the autocorrelation of observations is clearly a subject of primary analytical interest, based on the fact that measures in a close spatial proximity tend to be more similar than others spatially separated and on the basic necessity to answer the “how much is where” question of georeferenced data [11]. Methodologically speaking, if spatial effects exist but are not accounted for in a model, the resulting estimates may be unreliable [12]. The Bayesian version of this kind of models is a flexible and robust approach to situations where both explanatory variables and spatial correlation must be considered [13].

Literature counting with a geographic component dedicated to the associations between weight and socioenvironmental factors has been growing recently [14]. Examples targeted specifically to children can be found in both developed and developing countries [15–18], with the limitation of being generally oriented to just one simultaneous categorical response (overweight, obesity or their combination). Even in restricted cases in which several nutritional states were contemplated [19, 20], overweight and obesity were not included as separated conditions, although, their relationships with certain determinants, their health implications over overall health and consequent interventions can differ. In Costa Rica, this distinction is present in the current approach to non-communicable diseases [21]. Gamboa et al. [22] already examined both conditions individually and their link with one socioeconomic status variable in this country, yet, spatial effects were not taken into account.

In this study, a Bayesian multinomial model was built using aggregated Census data to contribute to the understanding of overweight and obesity district prevalence as separate conditions for school-aged children in Costa Rica, by exploring their associations with certain socioeconomic characteristics and their respective geographic distributions and exceedance probabilities with respect to national health goals.

## Methods

### Study region

Costa Rica is a country in Central America, bordered by Nicaragua, Panama, the Caribbean Sea, and the Pacific Ocean. It has a land area of 51,180 $\text {km}^2$ administratively divided into seven provinces, 82 cantons and 489 districts. A population of about 5.1 million people resides in this territory; an estimated of 72.8% in urban areas [23]. It is considered an upper middle-income country, characterized by solid human development indicators, among them, one of the lowest poverty rates in Latin America [24], but also by its unsuccessful efforts to reduce it during the last 25 years, not to mention an extremely high inequality, with a Gini index of 47.8 [25]. Costa Rica is also characterized by its almost universal health care coverage and its historic investment in health promotion and prevention strategies. The Ministry of Health is the entity responsible for the overall stewardship of the health system with a focus on health promotion. It is in charge of strategic planning, sanitary regulation, research, and technology development. The Costa Rican Social Security Fund (CCSS) is the entity that provides health insurance for the entire population of the country, through a first, second, and third levels of care [26].

### Data sources

The primary source of information is the Weight and Size Census 2016 [27]. This initiative captured anthropometric data of 347,379 children between 6 and 12 years old attending to the Costa Rican private and public scholar system in that specific period, disaggregated by administrative geographical units; provinces, cantons and districts. The districts were chosen as unit of analysis in order to increase the granularity and the representation of the socioeconomic heterogeneity at this level of aggregation [28]. Metrics from the 2011 Census [29], were associated to each spatial unit. As a result of this process, 472 territories were considered. Their basic characteristics in terms of area and general and interest population were summarized in Table 1.

**Table 1:** Descriptive statistics of basic spatial units attributes

Attribute [unit]	mean	sd	min	$\text {Q}_{1}$	median	$\text {Q}_{3}$	max
Overall population [persons]	9113.8	9590.3	273.0	2797.8	6097.5	11170.0	71384.0
Area [$\text {km}^{2}$]	108.2	188.2	0.5	8.7	35.2	141.7	2223.3
Children population [persons]	740.7	788.1	4.0	223.0	505.0	740.7	6102.0

Number of observations (districts): 472. Children population contains 3 missing values.

Overall population data obtained from the 2011 Census.

Children population refers to children measured in the Weight and Size Census 2016

### Study variables

**Fig. 1:**
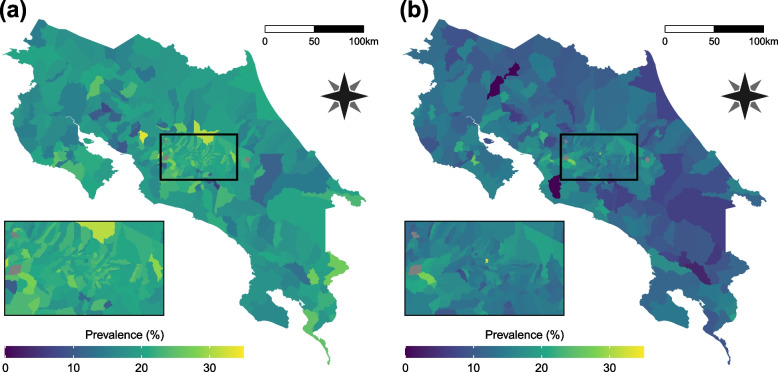
Geographical patterns of overweight and obesity prevalence in Costa Rica. **a** Overweight prevalence. **b** Obesity prevalence. Grey polygons represent districts without data

Overweight and obesity prevalence are the variables of interest in this study. They are defined as the percentage of children which body mass index (BMI), place them between 1 and 2 standard deviations above median (overweight) or more than 2 standard deviations above median (obesity) according to the WHO grow reference data [30]. Figure 1 presents spatial patterns of each of these conditions. The Empirical Bayes Index (EBI) [31], a modification of Moran’s I, was used to assess their spatial autocorrelation. The resulting values are 0.354 and 0.482 for overweight and obesity respectively, in both cases statistically considerable (*p*-value < 0.001).

The socioeconomic covariates that according to the literature could affect the prevalence values and were completed and available at district level are:

- Unemployment rate (*Unemployment*): in general, parental unemployment appears to be associated with the promotion of unhealthy dietary and activity behaviors, nevertheless, these associations depend on the studied country and whether unemployment comes from the mother or the father [32].
- Percentage of urban population (*Urban Population*): children living in urban areas are more likely to be overweight or obese than those in rural areas [33]. Urbanization is one of the most important drivers of change in dietary patterns and physical activity; the shortening of daily routes and the lack of open spaces for outdoor recreation tend to induce a sedentary lifestyle in children. [34].
- Percentage of homes with at least one critical deprivation (*Deprivation*): responds to the unsatisfied basic needs methodology proposed by the Economic Commission for Latin America (CEPAL). It establishes four critical aspects: access to dignified shelter, healthy life, knowledge and other goods and services [35]. The lack of one of the features is a poverty proxy. In developing countries, economic improvement is related to higher obesity levels in children [36, 37].
- Percentage of population under 14 years old (*Population*<14): Although the explicit link between this metric and childhood obesity was not found in the literature, there is evidence that greater presence of peers increase the physical activity in children and adolescents [38], a key factor in obesity reduction [39].
- Percentage of homes supported by a single mother (*Single Mother Homes*): single-parent households are positively related to higher levels of obesity and obesogenic behaviors in children [40]. In the case of single mothers, studies suggest that this can be attributed to non-sufficient income to provide high nutritional value food or access to organized physical activities, besides restricted parental time availability to monitor their diet and exercise habits [41]. It is important to note that most of the research in this area is limited to high income countries.
- Average number of occupants per home (*Occupants*): applying the quantity-quality trade-off theory, a nutrition quality decline will be expected as a consequence of a family size increase [42], however, larger families can also improve their nutrition due to the tendency to prepare home meal to take advantage of economies of scale [43]. Empirical results are also ambiguous and the studies so far are again limited to developed countries.
- Average years of schooling (*Schooling*): divergent behaviors describing the connection between obesity and educational attainment suggest the possibility of a non-linear relationship [44].

**Fig. 2:**
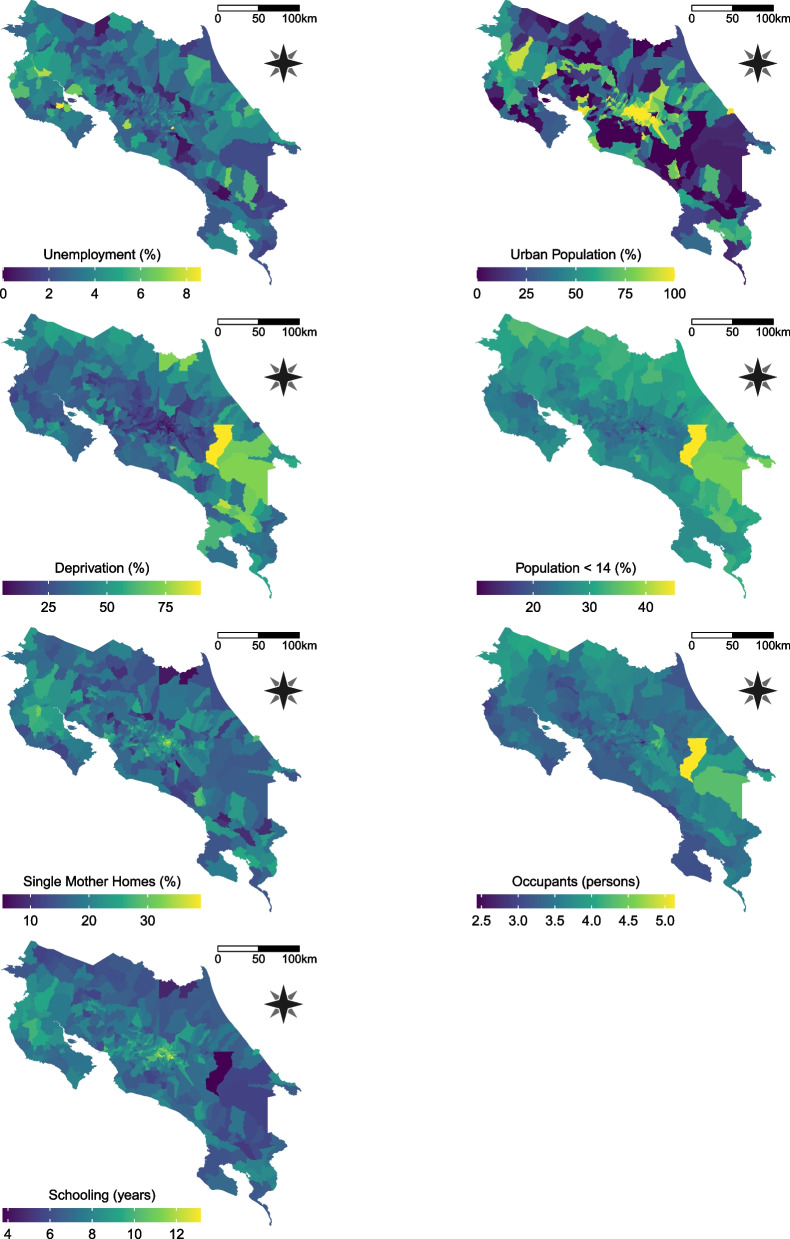
Geographical patterns of socioeconomic covariates

**Fig. 3:**
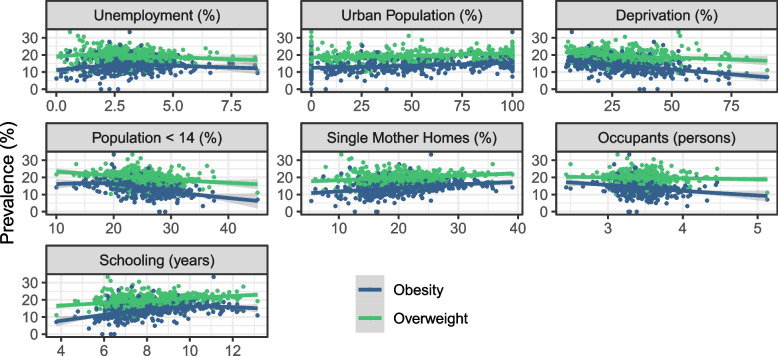
Association between covariates and prevalence of obesity and overweight

The spatial distribution of the values for each of the covariates is in Fig. 2 and their association with the incidence of obesity and overweight is shown in Fig. 3. Most of the associations are clearly linear but not necessarily considerable (e.g., *Urban Population*). It is important to note the concavity in the association between *Schooling* and obesity, supporting a possible existence of a non-linear relationship.

### Spatial model

For the *i*th district, let us consider the counts of children with overweight ($y_{i1}$), obesity ($y_{i2}$) and without any of these conditions ($y_{i3}$), as realizations of a vector of random variables $\mathbf {Y_{i}} = \{Y_{i1},Y_{i2},Y_{i3}\}$ with assigned probabilities $\mathbf {p_{i}}=\{p_{i1},p_{i2},p_{i3}\}$, under the restriction $\sum _{k=1}^{3} p_{ik} = 1$. If $M_{i}=\sum _{k=1}^{3} Y_{ik}$ then

1 $$\begin{aligned} Y_{i} \sim Multinomial(M_{i},\textbf{p}_{\textbf{i}}) \end{aligned}$$

The above model can be treated as a set of independent binomial logistic models where one of the categories acts as reference or baseline [45]. In this particular case, the baseline corresponds to the category of children without the conditions, resulting in:

2 $$\begin{aligned} \log {\Bigg (\frac{p_{ik}}{p_{i3}}\Bigg )} = \alpha _{k} + \textbf{x}_{i}^{\top } \varvec{\beta }_{\varvec{k}}, \quad k = 1,2. \end{aligned}$$

where $\alpha _{k}$ is the intercept, $\varvec{\beta _k}$ is a vector of fixed-effects and $\textbf{x}$ is the vector of the covariate values. Notice that due to the restriction $\sum _{k=1}^{3} p_{ik} = 1$, the probability for each condition and district can be expressed as a softmax function [46]:

3 $$\begin{aligned} p_{ik} = \frac{\exp (\beta _{k}\textbf{x}_{i})}{\sum _{k = 1}^{3}\exp (\beta _{k}\textbf{x}_{i})} \end{aligned}$$

A random effect $b_{i}$ can be incorporated in the model, adding a new element to each linear predictor to take into account additional variation or spatial correlation due to not contemplated factors:

4 $$\begin{aligned} \log {\Bigg (\frac{p_{ik}}{p_{i3}}\Bigg )} = \alpha _{j} + \textbf{x}_{i}^{\top } \varvec{\beta }_{\varvec{k}} + b_{i}, \quad k = 1,2. \end{aligned}$$

Using a BYM2 model [47, 48], the random effects corresponding to the *m* districts $\textbf{b} = \{b_{1},\dots ,b_{m}\}$ is defined as:

5 $$\begin{aligned} \textbf{b} = \frac{1}{\sqrt{\tau }}(\sqrt{1-\phi } \textbf{v} + \sqrt{\phi } \textbf{u}_{\varvec{*}}), \end{aligned}$$

Here, $\textbf{v} \sim \mathcal {N}(0,I)$ is an unstructured random effect, where *I* is the identity matrix of size *m*, and $\mathbf {u_{*}} \sim \mathcal {N}(0,Q_{*}^{-})$ is a scaled spatially structured component where $Q_{*}^{-}$ is the general inverse of the standardized version of the precision $m \times m$ matrix *Q*. Entries $Q_{ij}$ of the matrix *Q* are equal to the number of neighbors of district *i* if $i = j$, to $-1$ if *i* and *j* districts are neighbors, and 0 otherwise.

Finally, the term $\phi \in [0,1]$ is the proportion of the marginal variance explained by the latter effect and $\tau$ is the overall precision. These hyperparameters have Penalized Complexity (PC) priors [47], where $P(1/\sqrt{\tau } > U) = \alpha$ and $P(\phi < U) = \alpha$. In the case of $\tau$ the values for *U* and $\alpha$ were set to 0.5/0.31 and 0.01 respectively, while $U = 0.5$ and $\alpha = 2/3$ were used in the case of $\phi$, according to Simpson et al’s recommendation [47]. The fixed effects follows a normal distribution with mean 0 and precision 0.001.

All the available covariates were included as linear terms except for *Schooling*, that includes an additional quadratic term due to the behavior shown in Fig. 3. The estimation process was completed using Integrated Nested Laplace Approximation (INLA) through the R programming language [49] and the INLA package [50].

## Results

We considered several model alternatives (Table 2) based on an exploratory analysis where we examined variable descriptive statistics (Table 3), including their correlations (and potential multicollinearity issues), and their statistical and theoretical relevance. WAIC [51] and CPO [52] criteria were used for model selection, and since they provided similar values across models, the most parsimonious one was chosen. The final model includes

*Deprivation*, *Population*<14, *Schooling* and *Schooling*^2^ as predictors.

**Table 2:** WAIC and CPO values of the considered models

Model [Description]	WAIC	CPO
Model 1 [All variables]	6959.124	3597.787
Model 2 [Unemployment removed from Model 1]	6961.419	3599.480
Model 3 [ Occupants removed from Model 2]	6958.826	3597.954
Model 4 [Single Mother Homes removed from Model 3]	6959.543	3598.065
Model 5 [Urban Population removed from Model 4]	6959.926	3598.239

**Table 3:** Descriptive statistics of response and predictor variables including mean, standard deviation and correlations

Variable [unit]	mean	sd	(1)	(2)	(3)	(4)	(5)	(6)	(7)	(8)
Overweight [%] (1)	19.71	3.33	-							
Obesity [%] (2)	13.94	3.71	0.27	-						
*Unemployment* [%] (3)	3.07	1.23	-0.16	0.06	-					
*Urban population* [%] (4)	52.52	37.74	0.30	0.32	0.16	-				
*Deprivation* [%] (5)	29.29	13.90	-0.33	-0.45	-0.03	-0.69	-			
*Population*<14 [%] (6)	25.16	4.27	-0.31	-0.47	0.10	-0.48	0.74	-		
*Single Mother Homes* [%] (7)	20.21	5.48	0.23	0.31	0.26	0.69	-0.63	-0.55	-	
*Occupants* [persons] (8)	3.47	0.27	-0.07	-0.25	-0.01	-0.09	0.33	0.59	-0.31	-
*Schooling* [years] (9)	8.09	1.55	0.34	0.39	0.02	0.75	-0.84	-0.77	0.74	-0.41

Number of observations (districts): 472. Response variables contain 3 missing values

Table 4 contains estimates of these fixed effects. These estimates can be interpreted in terms of the change in the odds of overweight (or odds of obesity) relative to non-overweight and non-obese children. The third and fourth columns for each category present the 95% credible interval of the corresponding odds ratio.

**Table 4:** Fixed effect estimates in exponential scale. Posterior means, standard deviations, and lower and upper limits of 95% credible intervals

Variable	Overweight				Obesity			
	mean	sd	$\text {P}_{2.5}$	$\text {P}_{97.5}$	mean	sd	$\text {P}_{2.5}$	$\text {P}_{97.5}$
Intercept	0.204	0.062	0.112	0.369	0.200	0.081	0.091	0.442
Deprivation	0.998	0.001	0.995	1.001	0.992	0.002	0.988	0.995
Population<14	0.995	0.003	0.988	1.001	0.985	0.004	0.976	0.994
Schooling	1.130	0.062	1.014	1.259	1.189	0.087	1.029	1.373
$\text {Schooling}^{2}$	0.994	0.003	0.988	0.999	0.989	0.004	0.982	0.997

For both conditions, the credible intervals for *Schooling* and $Schooling^2$ confirm the hypothesized non-linear nature of the variable. Prevalences are positively related to the average of completed years of education until a certain point (approximately 8 years), then the relationship shifts.

In the case of *Deprivation* and *Population*<14, these variables are statistically relevant just for obesity, and suggest that districts with less affluent and younger population are associated  with lower odds of reaching this state. Particularly, there is a decrease of 7.7% ($[0.992^{10}- 1] \times 100$) in the relative odds of obesity when there is a 10% increase in *Deprivation*. Moreover, there is a decrease of 14% ($[0.985^{10} - 1] \times 100$) in the relative odds of obesity when there is a 10% increase in *Population*<14.

In Table 5 we included posterior estimates statistics of the hyperparameters in equation (5), where we can observe that, for both conditions, the posterior behavior of $\tau$ produces a non-trivial random effect and $\phi$ supports the existence of a structured spatial component.

**Table 5:** Hyperparameter estimates. Posterior means, standard deviations, and lower and upper limits of 95% credible intervals.

Hyperparameter	Overweight				Obesity			
	mean	sd	$\text {P}_{2.5}$	$\text {P}_{97.5}$	mean	sd	$\text {P}_{2.5}$	$\text {P}_{97.5}$
$\tau$	68.538	10.961	49.372	92.426	26.533	3.538	20.137	34.018
$\phi$	0.844	0.094	0.621	0.973	0.944	0.046	0.825	0.995

**Fig. 4:**
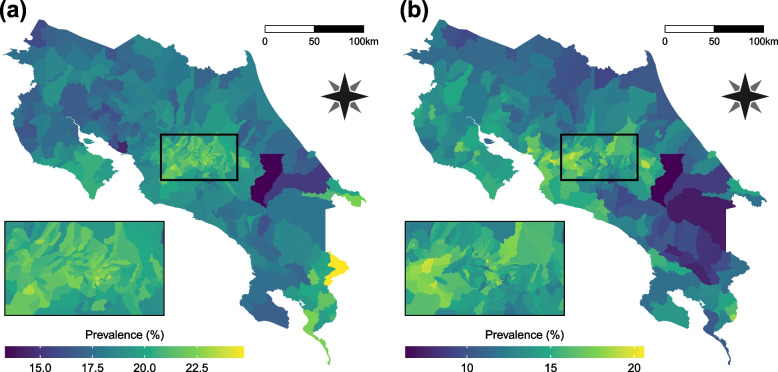
Geographical patterns of the estimated posterior mean prevalence values in Costa Rica. **a** Overweight estimated prevalence. **b** Obesity estimated prevalence

**Fig. 5:**
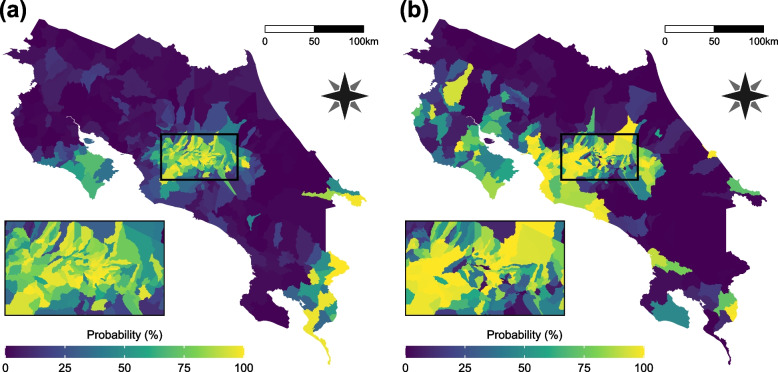
Geographical patterns of exceedance probabilities in Costa Rica. **a** Probability that overweight prevalence exceeds 20%. **b** Probability that obesity prevalence exceeds 14%

Figure 4 shows the posterior distributions mean values at each district per category. For overweight, higher values are concentrated in the center area of the country and in the border crossing areas to Panama. Obesity shows a more uniform pattern, but there is an evident contrast between the center of the country and the southeast districts that are not connected to commercial borders. A clearer picture can be seen in Fig. 5 which presents the exceedance probabilities, this means the probabilities of surpassing a given threshold, in this case, the current national values for overweight (20%) and obesity (14%), both established as baselines in the public health strategies directed to the study population [21]. The map in Fig. 5 (a) displays the probability that the overweight prevalence reaches 20% or higher in each district. Analogously, Fig. 5 (b) shows the probability of achieving a 14% or greater obesity prevalence. Besides the patterns already explained, districts with higher probabilities appear in the extreme of the upper peninsula (*Península de Nicoya*) in both maps, and in the Central Pacific coast in the case of obesity. These areas are connected by maritime transportation, creating a touristic and commercial route that changes the socioeconomic environment and consequently may affect the nutritional behavior of the residents. A similar explanation can be applied to the districts over the lower peninsula which have higher values relative to their surroundings. They receive the maritime traffic and are strategic stopover points before reaching the Panama border. Another detail to notice is that all administrative districts in the peripheral provinces present higher probability values. Again, this can be a consequence of the distinct characteristics of this localities, which concentrate governmental, health and recreational services.

### Dashboard

As an additional contribution to the Costa Rican health authorities, we developed a Shiny [53] dashboard showing the data and results obtained in this study. The dashboard contains maps and tables with the completed district database and their posterior distributions, which allows the user to calculate customized credible intervals and exceedance probability scenarios for overweight, obesity and combined prevalence, which consider the children with either of the conditions. The dashboard can be accessed at https://manjagoc.shinyapps.io/chovobcrdsh/.

## Discussion

With the progression in the prevalence of childhood overweight and obesity, public health authorities around the world are increasingly searching for innovated and locally adapted tools to better understand and address the diverse and complex factors leading to these conditions, including those social determinants that exceed the control of the children, their families, or their communities.

In this article, with the use of a Bayesian spatial mixed model, we analyzed geographic patterns of childhood overweight and obesity in Costa Rica and their links with some socioeconomic indicators. Given that multiple variables have been associated as risk factors of this increasing worldwide epidemic [54], our analysis allowed us to identify some of the ones that considerably affected the outcome of our model, as possible key drivers for childhood overweight and obesity in the country.

As it was suggested, the district average years of schooling proved to have a non-linear relationship with an increased weight status prevalence, especially with obesity. Results showed that a higher education, usually associated with an increased socioeconomic status, is initially related with augmented BMI in children, however, as educational levels become higher this relationship reverses and the prevalence starts to drop. These phenomenon have been identified in other studies, where education acts a “social vaccine” against obesogenic environments [55, 56] promoting a change in individual actions and healthier choices [57, 58]. At macro scale, with income as mediator, the same behavior has been detected in the relationship between economic development and obesity, defining what is known as the “Obesity Kuznest Curve” [59, 60].

In the case of the percentage of homes with at least one critical deprivation variable, the association was negative, implying that in districts with a wealthier population the odds of obesity in children increase, as observed in other studies from developing countries, where children born in a higher socioeconomic status have a higher risk of being overweight or obese, the opposite being observed in developed countries [61].

These results demonstrate the complex relationship between education, economic status and BMI in children, and highlights the importance of including public health interventions that target the promotion of healthy habits in parents at the forefront for the prevention of obesity, as their eating behavior, attitudes and perceptions are determinant in shaping the weight status of their children [62, 63].

The other variable that was statistically relevant for obesity was the percentage of population under 14 years old. The results suggests that districts with a higher proportion of older residents are associated with an increased likelihood of childhood obesity. This finding is consistent with previous research indicating that spending more time with friends may play an important role in reducing the risk of childhood overweight/obesity, as they promote more active lifestyles [64]. This aspect becomes more important as over the past several decades, virtually all regions in the world have experienced fertility decline [65]. In Costa Rica, it is projected that by 2025 the main population concentration will be located in ages between 25 to 44 years, with an increase of those 65 years and more [66]. Therefore, primary prevention methods should also be aimed at educating the child and encouraging appropriate diet and exercise from a young age through adulthood as well as promoting the availability of safe places for children to encourage daily physical activity.

As for the geographical aspects, the study showed that there are high probabilities of exceeding current national values of overweight and obesity in districts in the central area of the country and border areas in the southeast region, besides other locations characterized by its intense commercial or touristic activity.

Childhood overweight and obesity are due to multiple causes: genetic factors, hormonal derangements, environmental influences, and lifestyle, converging in this public health problem that affects society as a whole. Therefore, developing effective strategies and interventions to address the issue at hand requires a collective effort and the cooperation of all stakeholders. Academia can play a crucial role in this effort by providing valuable insights to guide the use of available resources. This article is one such effort to contribute to this field, although further research is still needed.

### Limitations and strengths

As a cross-sectional ecological study, our results are restricted to the understanding of the associations between certain socioeconomic characteristics of a population and its overweight or obesity prevalence. Therefore, they are not intended to serve as evidence for individual level or causal inferences [67, 68], nor to explain the complete etiology of this states. Besides, potential sex differences and relationships between the model predictors were not considered. Both constitute new research opportunities along with the inclusion of the temporal dimension when new data become available.

At the best of our knowledge, this is the first application of a spatial multinomial Bayesian model to explore the associations between socioeconomic factors and childhood nutritional states considering overweight and obesity as separate conditions. It is also the first study that analyzes the spatial aspect of these conditions in Costa Rica at national level. Another interesting feature is the use of the BYM2 model and the calculation of the prevalence exceedance probabilities with respect to Costa Rican health policy goals and the design of a Shiny app to support other researchers and authorities.

## Conclusions

The study found a non-linear relationship (U-inverted) between the average years of schooling per district and the prevalence of overweight and obesity. In addition, a negative association was found between the prevalence of obesity and the percentage of households with at least one critical deprivation and with the percentage of population under 14 years of age. The spatial patterns indicate that districts in the central area and southeast borders of Costa Rica, as well as other districts with high levels of touristic and commercial activity or administrative importance, have higher prevalence values and a greater probability of exceeding national obesity and overweight targets.

## Data Availability

The data that support the findings of this study are available from the Health Ministry of Costa Rica but restrictions apply to the availability of these data, which were used under license for the current study, and so are not publicly available. Data are, however, available from the corresponding author upon reasonable request and with permission of the Health Ministry of Costa Rica.
